# Retropharyngeal Lymph Node Metastasis Diagnosed by Magnetic Resonance Imaging in Hypopharyngeal Carcinoma: A Retrospective Analysis From Chinese Multi-Center Data

**DOI:** 10.3389/fonc.2021.649540

**Published:** 2021-06-11

**Authors:** Changming An, Ying Sun, Susheng Miao, Xiaoduo Yu, Ye Zhang, Xiwei Zhang, Lili Xia, Shaoyan Liu, Zhengjiang Li, Junlin Yi

**Affiliations:** ^1^ Department of Head and Neck Surgery, National Cancer Center/National Clinical Research Center for Cancer/Cancer Hospital, Chinese Academy of Medical Sciences and Peking Union Medical College, Beijing, China; ^2^ State Key Laboratory of Oncology in South China, Collaborative Innovation Center of Cancer Medicine, Guangdong Key Laboratory of Nasopharyngeal Carcinoma Diagnosis and Therapy, Sun Yat-sen University Cancer Center, Guangzhou, China; ^3^ Department of Radiation Oncology, Harbin Medical University Cancer Hospital, Harbin, China; ^4^ Departments of Radiation, National Cancer Center/National Clinical Research Center for Cancer/Cancer Hospital, Chinese Academy of Medical Sciences and Peking Union Medical College, Beijing, China; ^5^ Departments of Radiation Oncology, National Cancer Center/National Clinical Research Center for Cancer/Cancer Hospital, Chinese Academy of Medical Sciences and Peking Union Medical College, Beijing, China; ^6^ Xinxiang Medical University, Xinxiang, China

**Keywords:** hypopharyngeal cancer (HPC), hypopharyngeal squamous cell carcinoma (HPSCC), retropharyngeal lymph node (RPLN), prognostic, magnetic resonance image (MRI), pyriform sinus (PS)

## Abstract

**Background:**

To assess the prevalence, risk factors and prognostic significance of retropharyngeal lymph node (RPLN) metastasis diagnosed by magnetic resonance imaging (MRI) in patients with hypopharyngeal squamous cell carcinoma (HPSCC).

**Methods:**

259 patients from three cancer institutions in China from Jan 2010 to Dec 2018 were analyzed, retrospectively. All the patients had been given pre-treatment magnetic resonance imaging (MRI) of head and neck and were then treated with definitive radiotherapy with or without chemotherapy. Pretreatment diagnostic MRIs were reviewed by a dedicated head and neck radiologist, for the presence or absence of radiographically positive RPLN, cervical LN and tumor invasion.Demographic variables were analysed by descriptive statistics using SPSS 20.0. Predictors of the presence of RPLN and its prognostic significance were examined.

**Results:**

RPLN metastasis was discovered in 44 patients (17%). Logistic analysis showed that posterior pharyngeal wall (PPW) primary tumor; PPW invasion; N2-3; multiple cervical lymph node (LN) involvement (>2 LNs) were associated with RPLN metastasis, with metastasis rates 37%, 30%, 31% and 33% respectively. Patients with RPLN metastasis had a significantly reduced 5-year overall survival (OS) and disease-free survival (DFS) compared to the non-RPLN metastasis group (OS 28% vs. 48%, p=0.001; DFS 25% vs. 41%, p=0.040).

**Conclusions:**

RPLN metastasis was not uncommon in HPSCC patients. Risk factors were: PPW primary tumor, PPW invasion and cervical LN status. RPLN metastasis is a poor prognosticator for survival.

## Introduction

Among head and neck cancers, hypopharyngeal carcinoma (HPC) is most often detected at an advanced stage with poor prognosis, having 5 year survival rates of 31-47% (1–3). Due to the abundant lymph flow from the HPC, about 70% of patients have already presented with cervical node metastasis at their initial diagnosis (3). However, retropharyngeal lymph node (RPLN) metastasis of HPC often receives less consideration than metastasis to lymph nodes in the neck. There are few published reports concerning the incidence and role of RPLN metastasis in HPC (4, 5). Selected surgical reports have shown a direct pathway of drainage for hypopharyngeal cancers to the lateral retropharyngeal nodes through the ascending pathway, which can in fact bypass the jugulodigastric nodes (4, 6). However, the prevalence of RPLN involvement in HPC ranged hugely from approximately 10% to 60%, due to relatively small sample sizes (7).

The significance in prognosis of RPLNs metastasis in HPC is poorly understood. There is no consistency regarding the clinical significance of RPLN metastasis in HPC. Several studies suggested that RPLN metastasis significantly influenced overall survival and should be appreciated as an important prognostic factor in HPC (6). There are also a few studies which found no difference in local recurrence, distant metastasis or survival rates between the RPLN metastatic and RPLN non-metastatic groups (8).

Therefore, we investigated the treatment results in HPC from three centers in China, to estimate the prevalence of RPLNs in HPC, identify risk factors associated with RPLN metastasis and determine the prognostic implications of RPLN metastasis.

## Materials and Methods

### Patients

We retrospectively examined patients from Jan 2010 to Dec 2018 diagnosed with non-distant metastasis hypopharyngeal squamous cell carcinoma (HPSCC), from three cancers from China: Cancer Institute/Hospital, Chinese Academy of Medical Sciences, Peking Union Medical College; Sun Yat-sen University Cancer Center, the State Key Laboratory of Oncology in South China’s Collaborative Innovation Center of Cancer Medicine; and Harbin Medical University Cancer Hospital. Institutional Research Ethics Board approval was obtained prior to conducting the study.

### Evaluation of RPLNs and Treatment

All the patients had been given pre-treatment magnetic resonance imaging (MRI) of head and neck and were then treated with definitive radiotherapy with or without chemotherapy. Patients were excluded for the following reasons: unavailable pre-treatment MRI; previous head and neck cancer; or distant metastasis. Demographic, clinical, pathologic and radiologic data were reviewed for each patient. All the initial, pretreatment diagnostic MRIs were reviewed by a dedicated head and neck radiologist, for the presence or absence of radiographically positive RPLN, cervical LN and tumor invasion.

**Figure 1:**
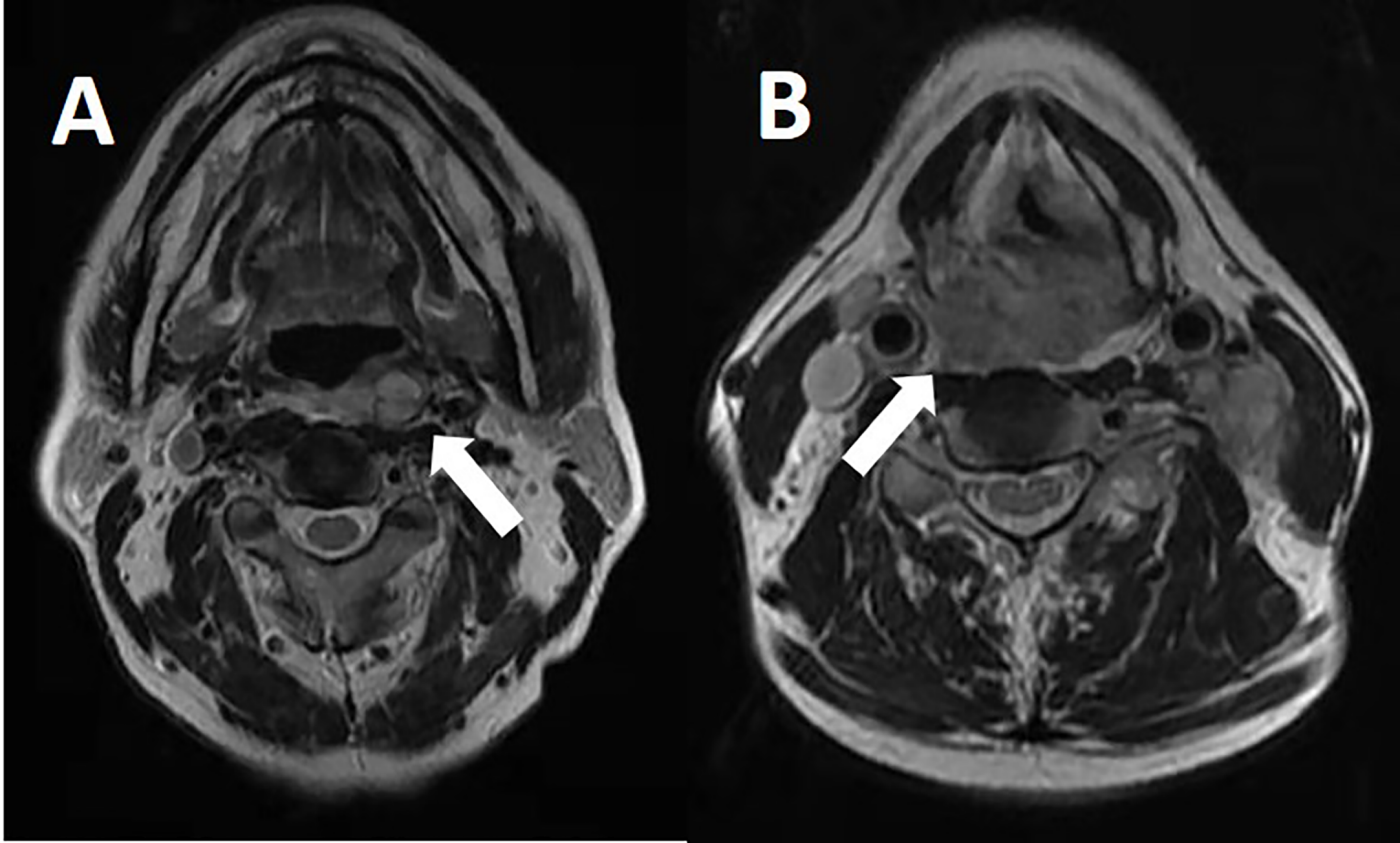
RPLN in MRI images. **(A)** Left RPLN metastasis in MRI. **(B)** Right RPLN metastasis in MRI.

Patients who met any of the following criteria were considered as having radiographically positive RPLN: in the axial plane, the largest short diameter of the retropharyngeal node ≥5mm, any visible median RPLN **(** **Figure 1** **)**, LN with circular enhancement or central necrosis;

For other cervical LNs, the largest short diameter of ≥10mm or ≥11mm at level II, a 3 LN grouping, each LN having a minimal axial dimension of 8-10mm; LN with circular enhancement or central necrosis; also LN with extracapsular spread. Information, including other variables, was also collected: this encompassed extranodal extension (ENE), matted nodes, the numbers of LN and the maximum diameter of cervical LN and RPLN.

The initial treatment was radiotherapy or concurrent chemoradiotherapy. The prescribed dose to the primary hypopharyngeal lesion was 66-70 Gy in 30 to 33 fractions. The positive lymph node was irradiated to 70 Gy in 33 fractions. The high-risk lymph node area including the retropharyngeal lymph node area and the neck level of positive lymph node received 60 Gy in 33 fractions. The low-risk lymph node area was administered 50 Gy in 28 fractions with the level and the next level below positive lymph node area. Cis-platinum was mostly in concurrent chemoradiotherapy.

Locoregional, failure-free survival (LRFS) was defined as survival without either emergence of primary site tumors or recurrence in the LNs. Distant metastasis-free survival (DMFS) was defined as survival without any clinical or radiographic evidence of disease outside of the head and neck region. Disease-free survival (DFS) was defined as survival without evidence of disease at any site, where both deaths and disease recurrence represented events. Overall survival (OS) was defined as death due to any cause. Treatment finish date was used as time point zero.

### Statistical Methods

Demographic variables were analysed by descriptive statistics using SPSS 20.0. Predictors of the presence of RPLN (gender, age, primary tumor invasion, N stage, ENE, matted nodes, numbers of LN, maximum diameter of LN) were examined in univariate and multivariate analyses using logistic regression.

LRFS, DMFS, OS and DFS were estimated using the Kaplan-Meier method. The significance of predictors of LRFS, DMFS, OS and DFS was assessed in univariate and multivariate analyses using COX proportional hazard models. A two-tailed p-value of less than 0.05 was considered statistically significant for all measures.

## Results

### Patient Demographics

A total of 259 patients diagnosed with HPC met our inclusion criteria; of these, 250 (96%) were male and 9 were female (4%). The median age was 57 years old (range: 36-85 years old). Tumor staging was reviewed according to the 7th edition of the UICC/AJCC TNM classification. Stage distributions were as follows: T1 in 32 cases, T2 in 94 cases, T3 in 95 cases and T4 in 38 cases. N stage distributions were: N0 in 75 cases, N1 in 55 cases, N2 in 119 cases and N3 in 10 cases. All patients were treated with definitive radiotherapy with or without systemic chemotherapy. Baseline clinical and treatment details can be found in **Table 1**.

**Table 1:** Baseline characteristics and treatment data of all the patients and those combined with RPLN metastasis.

Factors		Total (259)	RPLN+ (44)	Factors		Total (259)	RPLN+ (44)
Gender	Male	250	43 (17%)	Level II	Yes	146	37 (25%)
	Female	9	1 (11%)		No	106	7 (7%)
Age	≥50	194	33 (17%)	Level III	Yes	130	29 (22%)
	<50	65	11 (17%)		No	122	15 (12%)
Primary site	Pyriform sinus	206	26 (12%)	Level IV	Yes	35	6 (17%)
	Posterior pharyngeal wall	49	18 (37%)		No	217	38 (18%)
	Postcricoid region	4	0	NO. of LN	≤2	159	11 (7%)
Pharyngeal wall invasion	Yes	91	29 (32%)		>2	100	33 (33%)
	No	155	15 (10%)	Matted LN	Yes	77	27 (35%)
T stage	Not T4	164	17 (10%)		No	100	17 (17%)
	T4	95	27 (28%)	ENE	Yes	107	31 (30%)
N stage	N0-1	130	3 (3%)		No	70	13 (19%)
	N2-3	129	40 (31%)	Concurrent chemotherapy	Yes	139	24 (17%)
Clinical stage	I-II	37	0		No	82	15 (18%)
	III-IVa	222	44 (20%)		Unknown	38	5 (13%)

### Prevalence of RPLN Metastasis

Among the 259 patients, 44 patients (17%) presented with RPLN metastasis (the largest short diameter of the retropharyngeal node being ≥5mm in the axial plain). Among the 44 patients with RPLN metastasis, the median shortest size was 7mm (range 5-22 mm). 29 patients presented with unilateral RPLN metastasis and 15 patients with bilateral RPLN metastasis. Among the 29 patients who had unilateral positive RPLN, 25 patients demonstrated ipsilateral involvement, whereas 4 patients showed contralateral involvement. Stage distributions among the 44 patients with positive RPLN as the primary lesion were: T1 in 1 case, T2 in 12, T3 in 4 and T4 in 27 patients. Patient characteristics and treatment data are shown in **Table 1**.

### Risk Factors Associated With RPLN Metastasis

In univariate analysis all variables were tested, including gender, age, primary tumor status, nodal status, presence of ENE and presence of matted nodes. The relationship between RPLN metastasis and several clinical factors were analyzed. Univariate and multivariate analysis showed that independent factors associated with RPLN metastasis were: the primary tumor site being PPW vs. non-PPW (37% vs. 12%, HR=0.263, p=0.023); PPW invasion vs. no invasion (32% vs. 10%, HR=3.058, p=0.028); for N stage, N2-3 vs. N0-1 (31% vs. 3%, HR=0.106, p=0.008); and numbers of involved LN >2 vs. ≤2 (33% vs. 7%, HR=0.141, p=0.014). Univariate and multivariate analyses are shown in **Table 2**.

**Table 2:** Risk factors associated with RPLN metastasis in univariate and multivariate analysis.

Factors	Univariate analysis		Multivariate analysis	
	HR (95%CI)	*p*	HR (95%CI)	*p*
Gender	0.602 (0.073-4.937)	0.636		
Age	0.994 (0.470-2.101)	0.987		
Primary site	0.394 (0.212-0.735)	0.003	0.263 (0.083-0.833)	0.023
Pharyngeal wall invasion	4.366 (2.187-8.715)	0.000	3.058 (1.131-8.268)	0.028
T stage	0.291 (0.149-0.570)	0.000		
N stage	0.251 (0.144-0.439)	0.000	0.106 (0.020-0.564)	0.008
Level II	4.801 (2.047-11.260)	0.000	0.244 (0.063-0.947)	0.041
Level III	2.048 (1.038-4.043)	0.039	0.104 (0.025-0.443)	0.002
Level IV	0.975 (0.378-2.510)	0.957		
NO. of LN	0.659 (0.562-0.773)	0.000	0.141 (0.030-0.669)	0.014
Matted LN	2.637 (1.308-5.314)	0.007		
ENE	1.788 (0.857-3.723)	0.120		

### Prognosis Significance

**Figure 2:**
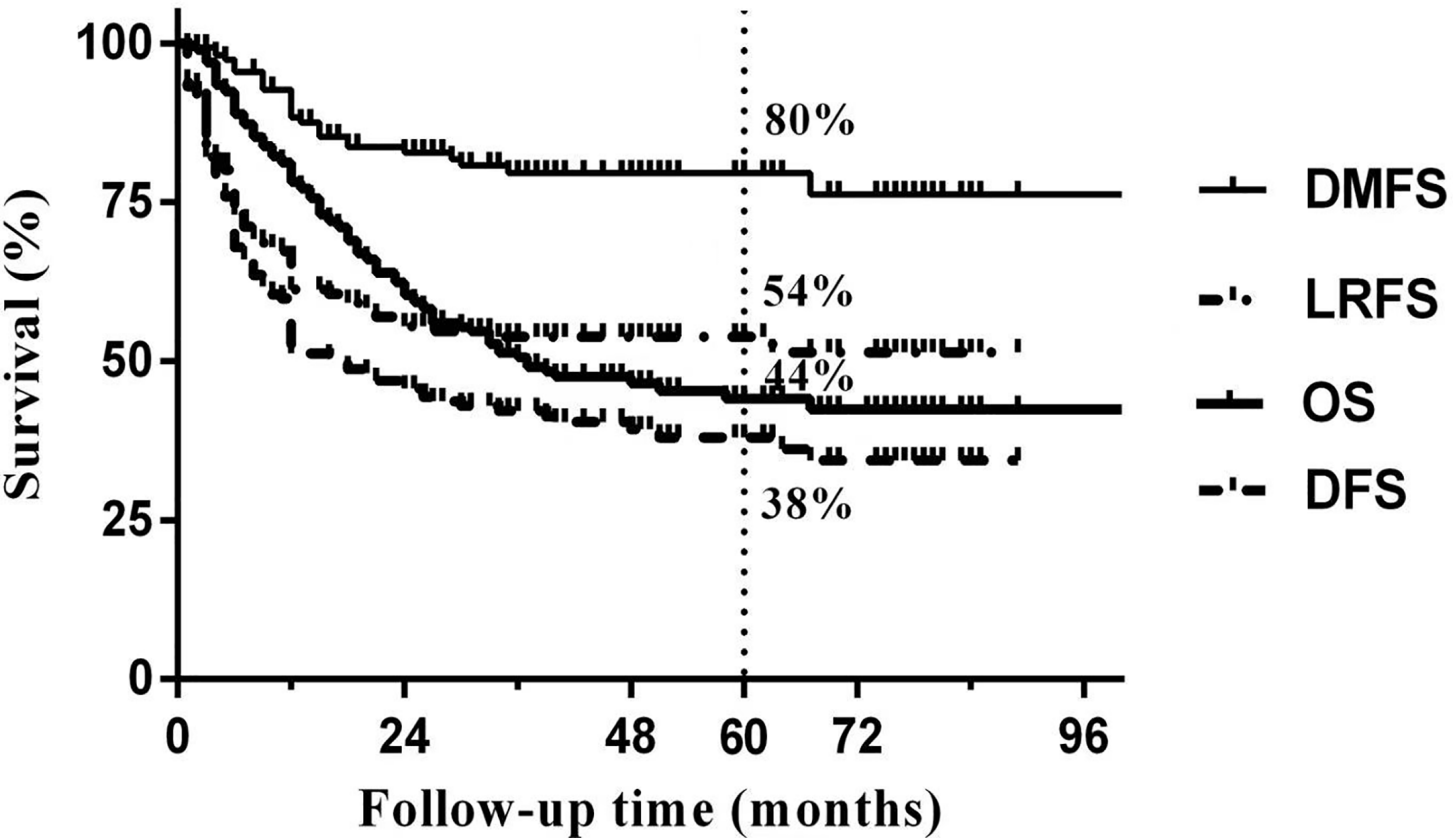
Survival curves in the whole cohort.

**Figure 3:**
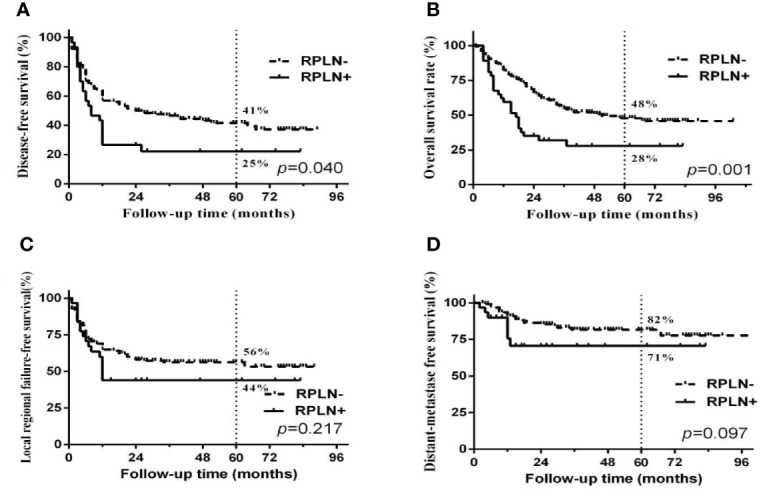
Survival curves between patients with or without RPLN metastasis. **(A)** disease-free survival; **(B)** Overall survival; **(C)** Locoregional failure-free survival; **(D)** distant-metastasis free survival.

Median follow-up time was 50 months (range 3 to 103 months). The overall 5-year DFS, OS, LRFS and DMFS rates in the whole cohort were 38%, 44%, 54% and 80%, respectively. Survival curves and failure patterns are shown in **Figure 2**. Patients with RPLN metastasis, when compared to the non-RPLN metastasis group, had significantly lower DFS (RPLN^+^ vs. RPLN^-^ at 25% vs. 41%, p=0.040) and OS (RPLN^+^ vs. RPLN^-^ at 28% vs. 48%, p=0.001). No significant difference was observed in DMFS (RPLN^+^ vs. RPLN^-^ at 71% vs. 82%, p=0.097) and LRFS (RPLN^+^ vs. RPLN^-^ at 44% vs. 56%, p=0.217) (**Figure 3**). Both univariate and multivariate analyses showed that age and RPLN involvement were independent prognostic factors associated with OS and DFS. The results of univariate analyses are shown in **Table 3** and multivariate analyses in **Table 4**.

**Table 3:** Results of univariate analysis in identifying factors associated with LRFS, DMFS, OS and DFS.

Factors		5y DFS		5y OS		5y LRFS		5y DMFS	
		%	p	%	p	%	p	%	p
Gender	Male	37	0.408	43	0.281	55	0.692	79	0.463
	Female	67		80		67		100	
Age	≥50	46	0.00	52	0.003	63	0.000	85	0.032
	<50	16		21		27		61	
Primary site	Posterior phayryngeal wall	35	0.453	47	0.853	56	0.772	79	0.564
	Not PPW	39		43		54		80	
Pharyngeal wall invasion	Yes	25	0.014	39	0.080	45	0.132	74	0.106
	No	43		45		57		83	
T stage	Not T4	44	0.040	44	0.931	58	0.124	83	0.220
	T4	29		46		47		74	
N stage	N0-1	49	0.004	57	0.004	66	0.002	78	0.664
	N2-3	29		33		42		81	
Level II	Yes	28	0.008	36	0.038	45	0.024	77	0.632
	No	53		56		64		82	
Level III	Yes	37	0.473	38	0.060	51	0.559	83	0.219
	No	39		52		54		75	
Level IV	Yes	32	0.211	21	0.071	41	0.207	80	0.760
	No	39		48		54		79	
No. of LN	≥2	43	0.020	52	0.007	60	0.024	78	0.812
	<2	33		35		45		81	
RPLN	Yes	25	0.004	28	0.001	44	0.217	71	0.097
	No	41		48		56		82	
Matted LN	Yes	31	0.616	32	0.140	45	0.632	67	0.040
	No	29		39		45		89	
ENE	Yes	37	0.305	37	0.612	50	0.465	83	0.129
	No	16		35		38		65	
Concurrent chemotherapy	Yes	40	0.699	50	0.093	54	0.909	85	0.086
	No	35		36		53		72	

**Table 4:** Results of multi-variates analysis in identifying factors associated with LRFS, DMFS, OS and DFS.

		HR	95CI	p
5y-DFS	Age	0.449	0.292-0.692	0.000
	RPLN	0.598	0.394-0.907	0.016
	Level II	0.558	0.351-0.886	0.013
5y-OS	Age	0.517	0.337-0.793	0.002
	RPLN	0.531	0.326-0.865	0.011
5y-LRFS	Age	0.414	0.257-0.669	0.000
	N stage	1.992	1.207-3.286	0.007
5y-DMFS	Matted LN	2.726	0.999-7.444	0.050

## Discussion

Our study, to the best of our knowledge, presents the largest cohort of patients with HPC using MRI to identify RPLN involvement. The involvement rate of RPLN was 17% in our dataset. Primary tumor status and cervical lymph nodes were closely related to RPLN metastasis. Primary tumor location at the posterior hypopharyngeal wall or tumor invading the posterior hypopharyngeal wall was associated with RPLN metastasis rates higher than 30%. Advanced N stage and multiple LN metastasis were also associated with high risk of RPLN disease. RPLN metastasis is a very poor prognosticator of DFS and OS in HPC.

RPLN receive afferent lymphatic drainage from pharynx and other sites, with efferent drainage to the upper jugular lymph node chain (9). RPLN is regarded as the first lymph node station in nasopharyngeal carcinoma, with the prevalence of RPLN metastasis very high, at up to 60% (10). RPLNs are also described as a nodal bed that are at risk for spreading either oropharyngeal carcinoma or HPC (9). However, due to their deep anatomical location, surgical dissection is somewhat complicated (6). Therefore, radiological or MRI assessment is essential for diagnosis. To date, there have been few published data on prevalence of RPLN involvement in patients with non-nasopharyngeal head and neck cancer and the frequency of positive retropharyngeal nodes reported in HPC varies hugely, ranging from 10% to 62% (10–12). These highly discrepant findings across different reports were mainly ascribed to two reasons, one being that most studies included other head and neck carcinomas and had small sample sizes, and were not limited to HPC; another reason being that various imaging technologies, such as CT, MRI and (18)F-FDG PET had been used (5, 13). Actually, MRI has been shown to be superior to CT images for detecting metastatic RPLNs; MRI is considered the preferred method for assessing metastatic RPLNs, as a guide to physicians prescribing appropriate treatment (14). Considering the advantage of MRI in detection of RPLN involvement and the fact that (18)F-FDG PET is not always available, we therefore chose pre-treatment MRI as mandatory in identifying RPLN in HPC patients in our cohort.

RPLN metastasis is not uncommon in HPC, and was found in 17% of our cohort. In order to find the risk factors associated with RPLN metastasis in HPC, univariate and multivariate logistic regression analyses were conducted. Primary tumor status and LN status both significantly affected RPLN involvement. Primary tumor site location is a significant factor associated with RPLN metastasis; patients presenting with pyriform sinus (PS) showed RPLN involvement at 12%, but with posterior wall tumors, at 37%, in accordance with other studies. We also found that primary disease with PPW invasion is an independent risk factor which is associated with RPLN metastasis (PPW invasion, 32% vs no-PPW invasion at 10%, OR=3.058, p=0.028), which indicates that PPW invasion may affect LN drainage regardless of the primary tumor location. RPLN metastasis appeared to be significantly associated with N status, whether N2-3 disease (p=0.008), level II (p=0.04), or level III (p=0.002). Higher risk of RPLN involvement was also associated with LN involvement and multiple cervical LN (p=0.014). HPC at advanced N stage (N2-3) and multiple cervical LN involvement (>2 LNs) tended to develop RPLN metastases at rates higher than 30%, a figure validated by previous studies. There are differing views about the influence of cervical LNs on RPLN involvement, with some data suggesting that level V LN involvement is an independent predictor of RPLN involvement in HPC, but it needs to be interpreted cautiously (5). In our cohort, cervical LNs in level II/III instead of level V, were independent predictors for RPLN metastasis, as similarly found in a previous report (5, 15).

It has been recognized that the RPLNs are of major importance as foci of metastases in nasopharyngeal carcinoma. However, in HPC the prognostic and clinical role of RPLN disease remains controversial and improperly defined. Among head and neck cancers, HPC is most often detected at an advanced stage presenting poor prognosis, with a 5-year survival rate of 31-47% and where more than half of patients will develop disease failure (16). In this study, 5-year OS and DFS were 44% and 38%, respectively. Metastasis to RPLN is recognized as an important prognostic factor and indicates an unfavorable prognosis in head and neck cancers. Our results showed that RPLN metastasis presented with lower OS (HR 0.531, p=0.011) and DFS (HR 0.598, p=0.016). Even after multivariate analysis, RPLN metastases were still independently significant as a prognostic factor associated with poor survival. Therefore, identifying patients with high risk of RPLN metastasis is of great importance in clinical treatment decision making, especially for guiding elective LN irradiation.

For patients without RPLN metastasis it is still controversial as to whether radiotherapy in the RPLN area is a beneficial treatment. According to recent publications, the RPLN area is routinely defined as the radiotherapy target regardless of the clinical stage of HPC (17). However, the guidelines composed by Gregoire et al. proposed that treatment of RPLNs with prophylactic radiotherapy is not essential for HPC patients with N0 or N1 classification (16). From our results, patients with PPW disease or PS disease with PPW involvement were prone to develop RPLN metastasis, with a rate higher than 30%, and we would also have recommended that these patients be irradiated in their RPLN regions.

Our data were collected from three cancer institutions from China and included only patients with HPC; their pretreatment MRIs were used to assess the prevalence of RPLN involvement, then related risk factors and their prognostic value were identified. Several limitations should be raised. First, a caveat inherent in this study is that MRI findings may not be consistent with the original pathological examinations. Since dissection, or biopsy, of RPLN is currently difficult to perform, pathological criteria are insufficient to identify RPLN involvement, so patients may originally have been differently diagnosed (not by MRI data). Secondly, data collection was retrospective, and treatment modalities were undoubtedly different in the three institutions. Notwithstanding the above limitations, we believe the current analysis from multiple cancer centers presents a detailed description of RPLN involvement and its prognostic role in HPC, providing evidence for recommending prophylactic irradiation of RPLN in HPC. This treatment should be of great clinical value.

## Conclusion

The prevalence of RPLN involvement in HPC is 17%. Risk factors for RPLN metastasis were: primary tumor located in PW, tumor with PW invasion, advanced N stage, multiple LN involvements, and level II/III LN involvement. Patients who were RPLN-positive showed significantly lower DFS and OS than those without RPLN involvement. Prophylactic treatment by irradiation of RPLN should be beneficial to all HPC patients.

## Data Availability Statement

The original contributions presented in the study are included in the article/supplementary material. Further inquiries can be directed to the corresponding authors.

## Ethics Statement

The studies involving human participants were reviewed and approved by Ethics Committee, Cancer Hospital, Chinese Academy of Medical Sciences. The patients/participants provided their written informed consent to participate in this study.

## Author Contributions

CA, YS, and SM contributed equally to this work. CA statistical analysis and wrote paper. CA, YS, and SM collected the patient data. XY, YZ, XZ and LX statistical analysis and evaluate the MR. SL, ZL, and JY: supervision. All authors contributed to the article and approved the submitted version.

## Funding

Supported by the Non-profit Central Research Institute Fund of Chinese Academy of Medical Science (2019-RC-HL-004) AND Beijing Hope Run Special Fund of Cancer Foundation of China(No. LC2018L06).

## Conflict of Interest

The authors declare that the research was conducted in the absence of any commercial or financial relationships that could be construed as a potential conflict of interest.
